# Diagnostic Feature Reconstruction for Enhanced Single-Lead ECG Classification

**DOI:** 10.3390/s26102955

**Published:** 2026-05-08

**Authors:** Chenhao Qi, Yu Guo, Qiping Yang, Yichen Hu, Yuanyuan Chen, Qiuyun Fan, Kangyin Chen

**Affiliations:** 1 Department of Biomedical Engineering, Medical School, Tianjin University, Tianjin 300072, China; 2 State Key Laboratory of Advanced Medical Materials and Devices, Tianjin University, Tianjin 300072, China; 3 Tianjin Key Laboratory of Ionic-Molecular Function of Cardiovascular Disease, Department of Cardiology, Tianjin Institute of Cardiology, Second Hospital of Tianjin Medical University, Tianjin 300072, China

**Keywords:** single-electrocardiogram classification, deep learning, feature reconstruction, feature enhancement

## Abstract

**Highlights:**

**What are the main findings?**

- Feature reconstruction effectively transfers 12-lead diagnostics to single leads.
- The integration of feature reconstruction and cross-attention fusion increases single-lead signal discriminability.

**What are the implications of the main findings?**

- Bridging this diagnostic gap makes wearable ECGs highly reliable for daily monitoring.
- This strategy provides an adaptable paradigm for other limited-sensor medical domains.

**Abstract:**

While the standard 12-lead ECG is vital for cardiovascular diagnosis, its reliance on clinical settings hinders daily use. Wearable few-lead devices offer a practical alternative, yet this convenience comes at the cost of diagnostic capability due to reduced lead coverage. To bridge this informational gap and enhance single-lead ECG diagnostic performance, we propose a feature-reconstruction-based classification method for single-lead ECGs. It leverages a pre-trained 12-lead ECG model to extract representative features and guide the feature learning process for single-lead signals. A CNN–Transformer-based multi-scale feature extraction module is introduced for robust ECG feature extraction, followed by a transformer encoder-based reconstruction module to align single-lead features with more discriminative 12-lead representations. A cross-attention based feature fusion module subsequently integrates the reconstructed and original single-lead features to enhance classification performance. By focusing on feature reconstruction rather than signal reconstruction, our method effectively avoids the performance degradation typically caused by signal reconstruction errors and inter-lead redundancy, leading to superior classification outcomes. Evaluation on two public datasets demonstrates that our method enhances feature discriminability and improves single-lead ECG classification performance, confirming its robustness and practical potential.

## 1. Introduction

Cardiovascular Diseases (CVDs) are among the leading causes of death worldwide. According to data from the World Health Organization (WHO), the number of deaths attributed to CVDs globally exceeded 17.9 million in 2021, accounting for over 31% of the total deaths [1]. Arrhythmia is a common type of cardiovascular disease, characterized by one or more episodes of irregular heartbeats. Many forms of arrhythmia, such as atrial fibrillation (AF) and ventricular fibrillation (VF), are extremely life-threatening. Therefore, the timely detection and accurate classification of arrhythmia are of crucial significance for the prevention and treatment of heart diseases.

Electrocardiogram (ECG) has become one of the most commonly used tools in the clinical evaluation and diagnosis of heart diseases [2,3]. The 12-lead ECG, by virtue of its ability to depict the spatial distribution of cardiac electrical activity through six limb leads and six precordial leads, provides multi-perspective and multi-directional cardiac electrical signals. This enables clinicians to identify and diagnose cardiac abnormalities with higher accuracy. Despite its high diagnostic value, the widespread application of the 12-lead ECG is still constrained by practical operational limitations. The standard 12-lead ECG system is typically installed in hospitals or medical institutions, and its utilization requires operation by professional medical staff and the attachment of multiple electrodes [4]. Consequently, although the 12-lead ECG possesses unparalleled advantages in terms of diagnostic accuracy, factors such as its strong dependence on specific environments and inconvenience in operation have restricted its scope of application.

With the advancement of wearable technology, an increasing number of lightweight smart wearable devices have provided a feasible solution characterized by low cost and wide coverage [5,6,7]. Wearable ECG devices aim to enable patients to collect their own ECG data through simplified methods, but this comes at the cost of reducing the number of leads—typically resulting in single-lead ECG. However, single-lead ECG can only record cardiac electrical activity from a single perspective, lacking spatial complementary information among multiple leads. This makes it difficult to capture focal abnormalities, and its accuracy in identifying cardiovascular diseases is far lower than that of 12-lead ECG.

Generative artificial intelligence has advanced the development of models that reconstruct ECG signals for absent leads [8,9,10]. These models aim to predict a full 12-lead ECG from a subset of available leads. However, fewer leads, especially a single lead, provide limited information. As a result, the generated signals may be of low quality, which could compromise the reliability of ECG-based diagnostic systems. Rather than directly reconstructing all 12 leads at the signal level, a practical alternative is to operate in the feature space, which focuses on discriminative features and avoids the redundancy of raw 12-lead signals. The goal is to learn intermediate representations from a single lead that approximate the discriminative features of a full 12-lead ECG, thereby enhancing the performance of downstream classifiers.

To bridge the diagnostic performance gap between single-lead and standard 12-lead ECG systems, this paper presents a feature reconstruction based single-lead ECG classification network (SLFR-Net). The framework employs a CNN–Transformer module for multi-scale feature extraction from single-lead inputs, guided by discriminative features from a pre-trained 12-lead classification model. A transformer-based reconstruction module then aligns these features with the comprehensive 12-lead feature space. Finally, a cross-attention based feature fusion module integrates the original and reconstructed features, yielding a robust representation that significantly enhances diagnostic accuracy. The key insight is that by operating in the feature space, our method simplifies the reconstruction process and avoids the common limitations of signal-level reconstruction, demonstrating superior accuracy and stability over conventional single-lead models in most experimental scenarios.

Our contributions can be summarized as follows:

- We introduce a feature-level reconstruction paradigm that replaces conventional waveform-level methods for single-lead ECG classification. By operating in a semantic feature space instead of performing point-by-point fitting, our approach reduces complexity while better serving downstream classification objectives.
- We design a cross-attention feature fusion module that integrates original and reconstructed features. This module effectively balancing fine-grained details from the raw signal with high-level semantic information from the reconstructed features, enhancing the robustness of the final representation.
- Through experiments on two public datasets, we demonstrate that feature-level reconstruction consistently outperforms signal-level approaches across various lead configurations, providing a valuable insight for future research in resource-constrained ECG analysis.

## 2. Related Works

### 2.1. Deep Learning Based Electrocardiogram Classification

In recent years, deep learning has been widely applied in the automatic ECG classification of arrhythmias owing to its powerful feature extraction capability. Such methods typically employ complex network architectures capable of discovering latent feature correlations within input signals, significantly enhancing their performance on complex modeling tasks.

Convolutional Neural Networks (CNNs), with their strong ability to capture local morphological patterns such as P waves, QRS complexes, and T waves, have become the primary choice for automatic ECG classification. Kirany et al. [11] integrated feature extraction and classification modules into a unified learning framework, achieving efficient personalized heartbeat classification without manual feature engineering. Hannun et al. [12] proposed an end-to-end deep neural network capable of identifying twelve arrhythmia types—including atrial fibrillation and ventricular tachycardia—with diagnostic performance comparable to that of cardiologists.

Given the pronounced temporal characteristics and long-term dependencies of ECG signals, researchers have progressively introduced Recurrent Neural Networks (RNNs) and attention mechanisms to capture inter-beat sequential information and contextual correlations. The hybrid CNN–LSTM architecture proposed by Zihlmann et al. [13] effectively detected paroxysmal atrial fibrillation events, demonstrating the effectiveness of convolution–recurrent hybrid models in ECG sequence classification. More recently, Transformers have gained traction, using self-attention to capture complex dependencies within ECG data and showing state-of-the-art results [14,15,16].

Accurate interpretation of ECG signals requires feature extraction across multiple temporal scales, as physiological patterns range from fine-grained waveforms to longer-term rhythms. Furthermore, the optimal convolutional scale varies significantly with lead type and patient demographics. Studies [17] have demonstrated that even minor adjustments to a model’s receptive field can substantially impact time-series classification performance—a finding equally applicable to arrhythmia detection. Consequently, multi-scale feature learning has proven essential for high-precision ECG classification, with multiple studies [14,18] confirming its critical role in achieving robust detection accuracy.

Some researchers have observed that standard 12-lead ECGs contain considerable information redundancy—which can lead to model overfitting and limited generalization—and have demonstrated that reducing this redundancy can enhance model performance [19,20]. For instance, Ramirez et al. [21] analyzing inter-lead redundancy found that for certain cardiac conditions, using fewer leads did not compromise diagnostic accuracy and could even improve it. These findings offer practical value for channel-constrained applications, such as developing efficient or lightweight models for wearable ECG devices.

With the widespread adoption of wearable devices, classification studies based on few-lead ECGs have attracted increasing attention [22,23,24,25]. Mainstream wearable systems currently employ the lead I configuration [5,26,27]. Current studies have mainly focused on the detection of specific arrhythmias [28,29,30]. For example, Ma et al. [31] achieved an accuracy of over 90% in atrial fibrillation detection from continuous wearable ECG recordings based on xResNet50. Other studies have demonstrated that neural networks can also diagnose myocardial infarction from single-lead ECGs [32,33,34]. However, these methods are mostly restricted to identifying a single type of disease.

### 2.2. Few-Lead ECG Classification

There remains a significant performance gap between single-lead ECGs and multi-lead ECGs in diagnostic accuracy. This discrepancy mainly arises from the fact that single-lead ECGs record cardiac electrical activity from only one perspective and are essentially a local projection of the 12-lead ECG, thereby failing to fully capture the spatial distribution of cardiac electrical activity. To address this limitation, researchers have proposed various ECG reconstruction approaches.

Lee et al. [35] developed a generative adversarial network (GAN) based on R-peak alignment to overcome the inability of portable ECG devices with limited leads to obtain the clinically required precordial leads. In this method, the limb lead II is used as input, the one-dimensional ECG signal is first transformed into a two-dimensional image, and a conditional GAN is then employed to generate the target precordial leads—providing a feasible pathway to complement chest-lead information from a single-lead input. To directly generate a complete 12-lead ECG from a single-lead input, Seo et al. [36] proposed a GAN-driven multi-lead ECG synthesis framework, which takes the single lead I signal as input and synthesizes the remaining eleven leads in an end-to-end manner via a conditional GAN. Compared with the method of Lee et al., this approach eliminates the need to convert one-dimensional time-series data into two-dimensional representations, significantly simplifying signal preprocessing while producing higher-quality reconstructed outputs.

Some studies have further integrated reconstructed ECG signals into cardiovascular disease classification tasks. Joo et al. [37] proposed the EKGAN model, which employs a dual-generator architecture where a label generator guides the inference generator to learn key physiological features, thereby enhancing the physiological consistency and diagnostic applicability of the reconstructed signals. EKGAN reconstructs 12-lead ECGs from lead I input, achieving diagnostic accuracy comparable to that of cardiologists. Akshit Garg et al. [10] utilized an improved attention-based U-Net to reconstruct 10-s 12-lead ECGs from lead II signals and applied them to a 27-class cardiovascular disease classification task, achieving accuracy comparable to that of original 12-lead signals. Zhan et al. [38] directly validated the diagnostic value of reconstructed signals through comparative experiments: by combining a conditional GAN with an R-peak alignment strategy, they achieved an average correlation coefficient of 0.742 between the reconstructed and real 12-lead ECGs. In arrhythmia classification, the model using reconstructed signals achieved an accuracy of 0.74—significantly higher than that of the original single-lead model (0.71) and close to that of the original 12-lead model (0.81)—effectively bridging the diagnostic performance gap between the two.

## 3. Methods

**Figure 1:**
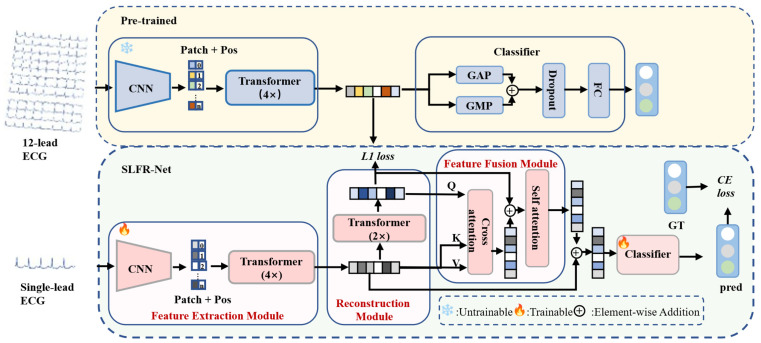
Architecture of the SLFR-Net. The pre-trained model consists of a feature extraction module and a classifier; the feature vectors output by the feature extraction module of the pre-trained model serve as the reconstruction targets for the reconstruction module of the SLFR-Net. The SLFR-Net consists of a feature extraction module, a reconstruction module, a feature fusion module, and a classifier. **Patch + Pos**: features with added position encoding; **GT**: the abbreviation for ground truth, representing the true label of samples in the dataset; **pred**: the model’s prediction.

As illustrated in Figure 1, the proposed method consists of two main components: a pre-trained 12-lead ECG classification model and SLFR-Net. A CNN–Transformer based multi-scale feature extraction network is used to extract discriminative features for both models. Within the single-lead pathway, a reconstruction module is incorporated to generate more discriminative features from the original single-lead representations. The reconstructed features are then fused with the original single-lead features for the final classification. The tensor size changes at the main stages of the model are summarized in Table 1. In the following subsections, we present the details of each module.

### 3.1. ECG Feature Extraction

ECG signals simultaneously contain local morphological features and global temporal features [39]. Local waveforms (such as P waves and QRS complexes) reflect fine-grained electrical activity, where subtle variations in amplitude, duration, and morphology often indicate specific cardiac abnormalities. At a broader level, ECG rhythms reveal longer-term patterns such as heart rate variability and arrhythmic sequences, reflecting system-level cardiac electrical behavior over extended durations. These rhythm-based features provide diagnostic information that is complementary to—and often cannot be fully captured by—local waveform analysis alone. Therefore, an effective feature extraction framework should integrate both local morphological and global rhythm-based representations to enable more accurate and comprehensive ECG interpretation. In this study, we designed a feature extraction module employing a CNN for local ECG features and a Transformer [40] for global dependencies.

**Figure 2:**
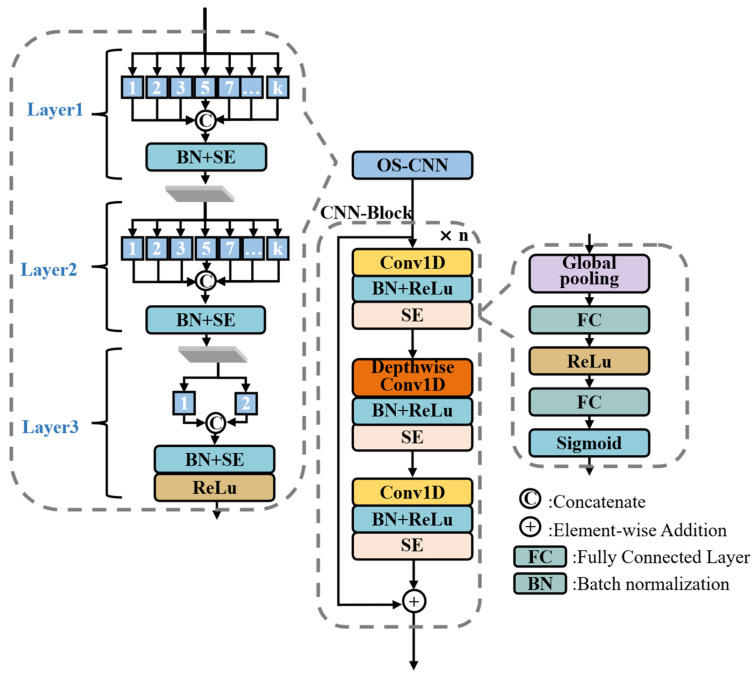
Detailed architecture of the CNN-based feature extraction, illustrating the **OS-CNN** and **CNN-Block** components. In OS-CNN, **1, 2, 3…k** represent the convolution kernel sizes, while **n** denotes the number of CNN blocks.

The Omni-Scale Convolutional Neural Network (OS-CNN) [17] is a convolutional neural network architecture based on multi-scale convolution, capable of extracting multi-scale features from ECG signals through convolution kernels of various scales. For ECG classification tasks, the optimal receptive field may vary across individuals due to physiological differences. Compared with conventional CNN, OS-CNN can more effectively adapt to diverse feature scales, thereby achieving a more suitable receptive field for ECG representation and classification. Therefore, this study employs OS-CNN as the first layer to capture multi-scale ECG representations. Additionally, this study integrates a Squeeze-and-Excitation (SE) [41] module into the OS-CNN, as shown in Figure 2, enabling the model to adaptively recalibrate the importance of multi-scale features.

Following OS-CNN, a CNN-Block is applied to streamline the extracted multi-scale ECG features, reducing dimensionality and refining the most discriminative information. The detailed structure of the CNN-Block is illustrated in Figure 2. After each convolutional layer in the CNN-Block, batch normalization and a ReLU activation function are first applied. The resulting features are then passed through an SE module, which can strengthen the feature representation. The first and third convolutional layers of the CNN-Block use a kernel size of 1, while the second layer utilizes a depth-wise convolution with a kernel size of 3. The first convolutional layer performs channel expansion, followed by deep convolution to implement spatial filtering. The final convolutional layer completes channel compression. This design effectively reduces computational complexity in subsequent operations while preserving key features.

Finally, a four-layer transformer module is applied after CNN-based local feature extraction to capture global ECG patterns. Through its self-attention mechanism, the model computes dependencies across all time steps, enabling it to recognize long-range physiological relationships—such as the progression from P waves to QRS complexes and T waves.

### 3.2. Feature Reconstruction from Single to 12 Leads

Unlike traditional methods that first fully reconstruct the 12-lead ECG waveform before classification, our approach directly reconstructs a discriminative 12-lead feature vector from the single-lead features.

The target 12-lead features are obtained from a frozen pre-trained model, which is trained on 12-lead data for a diagnostic task. The pre-trained model consists of the CNN–Transformer based multi-scale feature extraction module described in the above subsection and followed by a classifier. We define the multi-scale feature extraction module as *E_full_*, the forward process can be formulated as:

(1) $$F_{full}=E_{full}\left(X,\theta \right)$$

(2) $$P_{pre}=softmax\left(W_{pre}F_{full}+b_{pre}\right)$$

where *X* is the input 12-lead ECG, *θ* is the parameter of the *E_full_*, *F_full_* is the feature vector from the feature extraction module, *W_pre_* and *b_pre_* are the classifier’s weight matrix and bias, and *P_pre_* denotes the prediction. Once trained, this model provides discriminative feature representations that supervise the reconstruction of 12-lead features from their single-lead counterparts. The same feature extraction architecture is used for both 12-lead and single-lead inputs. The only difference lies in the number of input channels.

**Figure 3:**
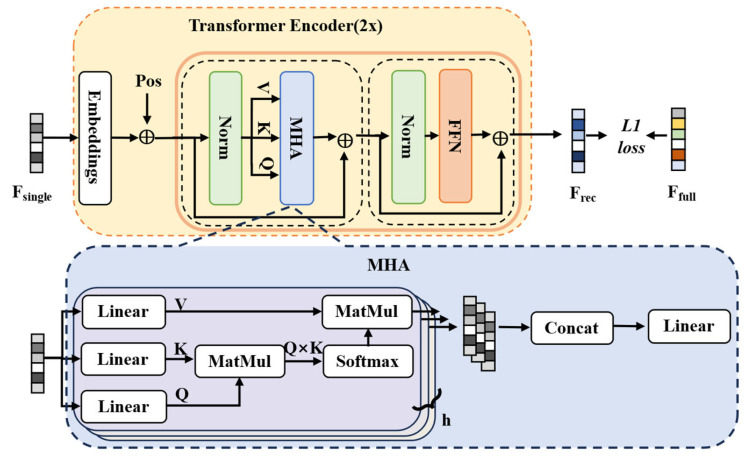
Architecture of the reconstruction module. **MHA** refers to multi-head attention and **FFN** refers to the feed-forward network, which are standard components of the Transformer. **MatMul** refers to matrix multiplication. **Pos** denotes position encoding.

This study introduces a transformer encoder-based framework, illustrated in Figure 3, to align single-lead ECG features with the more discriminative feature distribution of 12-lead ECG. The module uses a self-attention mechanism to learn a direct mapping from single-lead to 12-lead feature representations, capturing global dependencies without being limited by local receptive fields. The encoder is implemented with two layers to balance model capacity and computational efficiency. The features from the single-lead input, the target 12-lead features (extracted by the pre-trained model), and the reconstructed features are denoted as *F_single_*, *F_full_* and *F_rec_* respectively.

The reconstruction loss is measured using the L1 norm between the reconstructed features *F_rec_* and the target 12-lead features *F_full_*, formulated as:

(3) $$L_{rec}={\left\|F_{rec}-F_{full}\right\|}_{1}$$

The L1 loss is chosen for its ability to promote robust and stable feature learning. The L1 norm encourages the model to focus on reconstructing the most discriminative components of the feature representation, leading to improved alignment between the single-lead and complete 12-lead feature distributions. By optimizing this objective, the single-lead ECG-based classification network learns to produce feature vectors that closely match the discriminative characteristics of the 12-lead ECG signals.

### 3.3. Cross-Attention Guided Feature Fusion for Enhanced Single-Lead ECG Classification

After obtaining the single-lead feature *F_single_* and the reconstructed feature *F_rec_*, we combine them in a feature fusion module to leverage their complementary strengths. While *F_rec_* offers a more comprehensive representation, it may contain artifacts; conversely, *F_single_* is stable but informationally limited.

As shown in Figure 1, the feature fusion module consists of a cross-attention layer followed by a self-attention layer. In the cross-attention stage, *F_single_* is projected to key (*K*) and value (*V*) vectors, while *F_rec_* is mapped to the query (*Q*). The interaction between query and key is used to weight the value vectors, producing an initial fused representation:

(4) $$CrossAttn\left(Q,K,V\right)=softmax\left(\frac{QK^{T}}{\sqrt{d_{k}}}\right)V$$

This output is then passed through a self-attention layer to capture internal dependencies. It serves as an adaptive refinement mechanism that enhances informative features, suppresses redundant or noisy information, and enables the network to focus on important positions and discriminative components.

The proposed fusion strategy preserves both the robustness of the original signal and the enhanced discriminability of the reconstructed features. This enables the model to capture more complete electrocardiographic characteristics, improving both robustness and generalization under single-lead constraints.

The resulting representation is passed to a classifier, which applies global average pooling and global max pooling to the features, followed by a fully connected layer and softmax activation to produce the final prediction:

(5) $$P_{pred}=softmax\left(W_{pred}f_{\mathrm{enhance}}+b_{\mathrm{pred}}\right)$$

where *W_pred_* and *b_pred_* are learnable weight and bias parameters, and *F_enhance_* denotes the enhanced feature representation output by the feature fusion module. The classifier output demonstrates the improved classification performance achieved by the single-lead ECG analysis system.

### 3.4. Loss Function

Two types of loss functions are used in this study to supervise the training of the proposed network. They are L1 loss (*L*_1_) in Equation (3) and multi-class cross-entropy loss (*L_CE_*). The L1 loss constrains the reconstruction module to preserve discriminative features while avoiding over-smoothing. The multi-class cross-entropy loss measures the difference between the predicted distribution and the true label distribution. The total training loss (*L_Total_*) is defined as the weighted sum of the two components:

(6) $$L_{Total\;}=L_{1}+\;\alpha \times L_{CE}$$

where *α* is weighting coefficients that balance the contribution of each loss term.

## 4. Experimental Setup

### 4.1. Database

The SLFR-Net was evaluated on the two public datasets: CPSC2018 and CODE-15%, which are described as follows:

(1) The 2018 China Physiological Signal Challenge (CPSC 2018) [42] provides 6877 publicly accessible 12-lead ECG records from 11 hospitals. The recordings vary in lengths from 6 to 60 s and were sampled at a frequency of 500 Hz. The dataset covers nine types of heart rhythms, including normal rhythm and eight arrhythmia categories. In this study, only the primary diagnostic label was retained for each ECG record, and low-quality segments were excluded. The resulting dataset distribution is presented in Table 2.
(2) CODE-15% dataset [43] is a large-scale multi-class 12-lead ECG dataset, containing 345,779 samples. Each sample has a duration of 10 s and a sampling frequency of 400 Hz. This dataset comprises normal ECGs, six types of disease-labeled ECGs, and additional abnormal ones. To maintain task consistency, we used only single-label samples, including 30,000 randomly selected normal cases and all available uniquely labeled abnormal cases, resulting in 64,104 records for experimentation, as shown in Table 3.

### 4.2. Implementation Details

For both datasets, low-quality ECG recordings were first excluded, followed by preprocessing procedures including denoising and normalization. Specifically, incomplete recordings and those with significant amplitude anomalies were removed. In the preprocessing stage, ECG signals from the CPSC 2018 dataset were standardized to a fixed length of 20 s using zero-padding for shorter recordings and truncation for longer ones. For the training data, zero-padding was randomly distributed before and after the signal while keeping the total duration fixed at 20 s. For the non-training data, zero-padding was applied only at the end of the signal. All signals were then denoised using a third-order Butterworth band-pass filter (0.05–48 Hz) to suppress baseline wander and high-frequency noise, normalized using z-score normalization, and downsampled to 250 Hz to ensure consistency and improve computational efficiency.

The preprocessed data were then divided into training, validation, and test sets with a ratio of 7:1:2. The split was performed at the patient level to prevent data leakage. To address the problem of class imbalance, a class-balanced sampling strategy was used to randomly select samples during training. Although this strategy may lead to repeated sampling of minority class samples, it helps alleviate the impact of class imbalance on model training.

All experiments were conducted under the PyTorch 1.12.1 framework using two NVIDIA GTX 3090 GPUs (24 GB each). During training, the 12-lead model is first pretrained using 12-lead ECG data. After training, all parameters of this pre-trained model were frozen, and the SLFR-Net was trained on the single-lead ECG from the same dataset, while the feature vectors output by the pre-trained model were used to supervise the SLFR-Net. This experimental design maintains data consistency while preventing leakage between the two tasks. The network was trained for 120 epochs with a batch size of 128. The Adam optimizer was used for adaptive learning rate adjustment: when the loss did not decrease for 5 consecutive epochs, the learning rate was reduced to half of its previous value. The initial learning rate was set to 1 × 10^−4^, and the minimum learning rate was set to 1× 10^−7^.

### 4.3. Evaluation Metrics

Model performance was evaluated using several common metrics. Accuracy measures the ratio of correctly classified samples. Precision, recall, F1-score and AUC were computed using macro-averaging (averaging metric values across all classes) to reflect overall performance unbiased by class distribution.

## 5. Experimental Results

### 5.1. Results on the CPSC2018 Dataset

As summarized in Table 4, the proposed feature-reconstruction-based classification model demonstrates performance gains over the single-lead baseline across all metrics on lead I of the CPSC2018 dataset, with improvements exceeding 6% in accuracy, recall, and F1-score. Statistical significance analysis shows that these improvements are significant (*p* < 0.001), indicating that the observed gains are meaningful. These results validate the effectiveness of the feature reconstruction approach. Nevertheless, a performance gap remains when compared to the 12-lead model.

**Figure 4:**
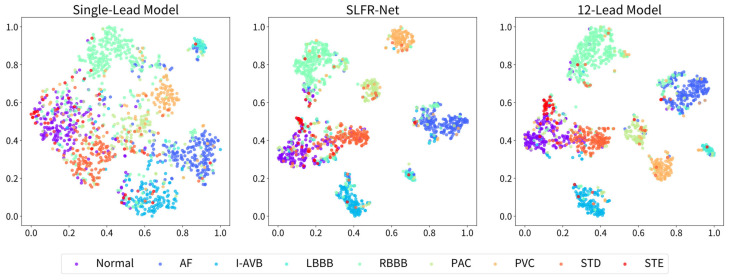
T-SNE plot of the feature distribution of single-lead model, SLFR-Net, and 12-lead model.

To intuitively illustrate the performance gap between the single-lead and SLFR-Net, we visualize the feature distributions using T-SNE graphs. As shown in Figure 4, the single-lead model exhibits varying degrees of feature confusion across categories, with the most severe overlap observed among normal, ST-segment depression (STD), and ST-segment elevated (STE) classes. In contrast, the SLFR-Net demonstrates sharper inter-class separation, with the feature distribution of each category showing noticeable improvement. This result indicates that the SLFR-Net effectively compensates for the diagnostic limitations of single-lead ECG in identifying specific arrhythmias.

**Figure 5:**
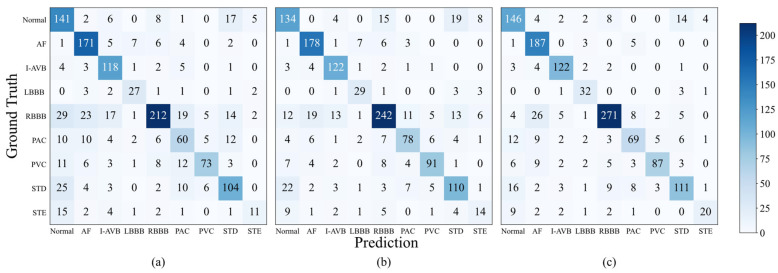
Confusion matrices of three models on the CPSC2018 dataset, (**a**) Single-lead model, (**b**) SLFR-Net, (**c**) 12-lead model.

**Figure 6:**
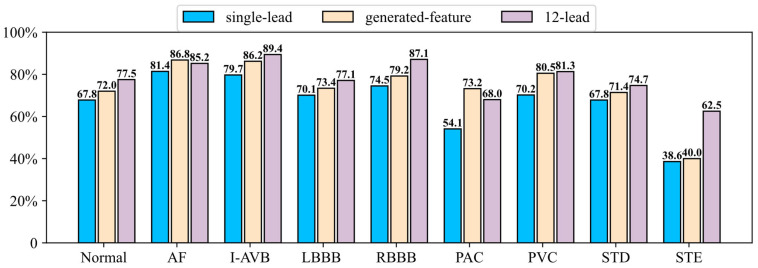
F1-score performance of different models across categories (CPSC2018 dataset).

The confusion matrix and per-category F1-scores are shown in Figure 5 and Figure 6, respectively. By comparing the results across different categories, it can be observed that the performance gap between the single-lead model and the 12-lead model was relatively small in the AF category, while larger gaps existed in other categories. The SLFR-Net improved classification performance across all categories compared to the single-lead baseline, demonstrating its ability to effectively recover discriminative information from lead I ECG. However, the performance gap with the 12-lead model persists, likely due to the inherent challenge of reconstructing certain critical features that are uniquely captured by multiple leads. For arrhythmias such as AF, Premature atrial contraction (PAC), and Premature ventricular contraction (PVC), which are characterized by clear electrophysiological features, the SLFR-Net achieved accuracy matching or exceeding that of the 12-lead model. This indicates that the reconstructed features effectively capture the essential diagnostic information, whereas the comprehensive 12-lead input may introduce redundancy that limits model efficiency in these specific tasks. Notably, both the single-lead model and the SLFR-Net showed poor performance in the STE category. This result may be attributed to the limited number of STE cases in the dataset, which provides an inadequate basis for the learning of its distinctive features.

### 5.2. Results on the CODE-15% Dataset

**Figure 7:**
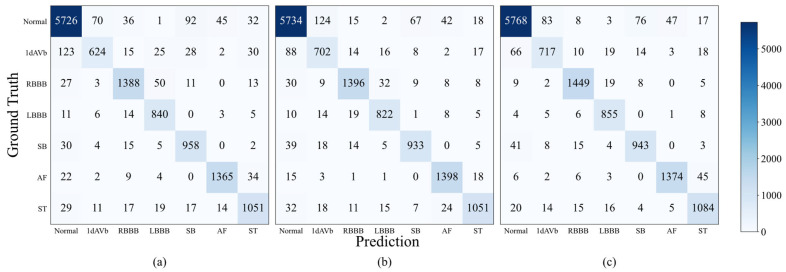
Confusion matrices of three models on the CODE-15% dataset: (**a**) Single-lead model, (**b**) SLFR-Net, (**c**) 12-lead model.

Compared with the CPSC2018 dataset, the CODE-15% dataset has a larger sample size and fewer categories. Consequently, the single-lead model, the proposed reconstruction model, and the 12-lead model all demonstrated better overall performance. As shown in Table 5, SLFR-Net improves all evaluation metrics compared with the single-lead model. Statistical significance analysis shows that these improvements are significant (*p* < 0.001), indicating that the observed gains are meaningful. Figure 7 presents the confusion matrices of each model. Similar to the results observed on the CPSC2018 dataset, the SLFR-Net enhanced classification performance for most categories. Meanwhile, with the increase in ECG data available for training, the SLFR-Net has been able to achieve performance comparable to the 12-lead model in multiple categories. This result further verifies the reliability of the feature reconstruction-based method and highlights its potential for application in general ECG classification tasks.

### 5.3. Comparative Experiments

We compared SLFR-Net with signal reconstruction-based methods and knowledge distillation (KD) methods on the CPSC2018 and CODE-15% datasets. Reconstruction-based methods first reconstruct 12-lead ECG from single-lead (Lead I) inputs and then perform classification. EKGAN [37] and multi-channel masked autoencoder (MCMA) [44] were trained under the same settings as this study to ensure a fair comparison. Results of GAN [38] were directly adopted from its original literature. Since GAN was not evaluated on the CODE-15% dataset in its original study, it was excluded from the comparison on this dataset. Two typical knowledge distillation methods were selected for comparison, including logit-based distillation and feature-based distillation, where the 12-lead model served as the teacher model and the single-lead model as the student model. Feature-based distillation is performed under two settings, using L1 loss and Kullback–Leibler (KL) divergence to supervise the intermediate features from the last four layers. The specific results are shown in Table 6 and Table 7 below.

Based on the comparative results on the two datasets, SLFR-Net performs better and reaches the best or near-best results across multiple metrics. On the more challenging CPSC2018 dataset, the feature reconstruction-based approach yields significant performance improvements. On the CODE-15% dataset, although the gap between single-lead and 12-lead models is small and the scope for improvement is limited, SLFR-Net still maintains competitive performance.

### 5.4. Ablation Experiments

To evaluate the contribution of each component, we conducted an ablation study on the CPSC2018 dataset. Results are shown in Table 8. The Baseline is the initial single-lead classification model, and all other models are derived from it by incrementally adding components.

**Effect of Pre-model Supervision**: First, we added a pre-trained model (trained using 12-lead ECG) to supervise the extracted features of the Baseline. Specifically, the features of 12-lead ECG are used to guide the feature learning of single-lead ECG. By continuously optimizing the L1 loss between these two types of features, the Baseline can learn richer and more discriminative feature representations, leading to gains in classification performance as shown in the results.

**Effect of Feature Reconstruction**: We next added a reconstruction module, changing the supervision target from the original single-lead features to the reconstructed output. This design enables the single-lead feature extraction module to focus on extracting single-lead-specific features, while the reconstruction module is specialized in generating features that mimic the richer 12-lead feature distribution, resulting in additional performance gains (F1-score improvement of 0.029).

**Effect of Feature Fusion**: The feature fusion module integrates the original single-lead features with the reconstructed 12-lead features, producing a richer, more comprehensive representation for subsequent classification. Experimental results confirm that this enriched representation further improves the final classification accuracy.

In addition to the module-level ablation studies, we also investigated the effect of the number of transformer decoder layers in the reconstruction module. The results are summarized in Table 9. Experiments show that using two layers yields the best classification performance.

## 6. Discussion

This section provides supplementary discussions on the impact of different feature reconstruction frameworks, loss functions on classification performance and the model’s effectiveness when applied to additional leads.

### 6.1. Impact of Feature Reconstruction Frameworks on Classification

This study employs a transformer-based encoder for the reconstruction of 12-lead features, leveraging its ability to integrate global information and generate well-structured representations from incomplete data. A CNN-based encoder–decoder architecture provides another common reconstruction framework. It effectively captures local spatial correlations, enabling the extraction of fine-grained signal details while preserving local continuity. Overall, CNN-based architectures focus on local structural representation, whereas transformer-based architectures emphasize global feature integration.

We evaluated the model’s performance using different reconstruction architectures. As shown in Table 10, the CNN-based and transformer-based reconstruction modules achieve comparable performance, with the transformer exhibiting advantages in recall, AUC, and F1-score. This performance gap may be attributed to the limited receptive field of the CNN, which constrains its ability to model long-range dependencies—a critical requirement for capturing diagnostic features spanning multiple heartbeats in ECG signals. Additionally, CNNs tend to prioritize local pattern and shape similarity, which may not align with the need for semantic consistency when reconstructing discriminative diagnostic features rather than replicating the original waveform.

### 6.2. Impact of Feature Reconstruction Loss Functions on Classification

In the reconstruction module, the choice of loss function directly defines the model’s learning objective by specifying how “similarity” is measured. To evaluate the effectiveness of different supervision strategies beyond the L1 loss, we compared the performance of L2 loss, cosine similarity (COS), KL divergence, and Jensen-Shannon (JS) divergence.

The experimental setup keeps the network structure and dataset division unchanged, modifying only the loss function of the reconstruction module. Different loss functions focus on different reconstruction goals: LI and L2 losses emphasize numerical point-wise proximity, cosine similarity loss assesses vector orientation consistency, and KL/JS divergences quantify the distance between probability distributions. Table 11 compares classification performance under different loss functions. The L1 loss achieves optimal results across all metrics, whereas the L2 loss yields comparatively poorer performance. Prior studies [45] suggest that the L2 loss is prone to local minima and imposes uniform Euclidean constraints, which may contribute to its suboptimal performance. In contrast, the L1 loss more effectively preserves local signal variations and subtle patterns, thereby enhancing the reconstruction of clinically critical diagnostic features. Although the cosine similarity loss and divergence-based (KL, JS) losses also performed well, they exhibit inherent limitations. The COS aligns vector directions while ignoring magnitude information, and its optimization inherently drives the growth of embedding norms, which can lead to amplitude mismatches between reconstructed outputs and original inputs [46]. The divergence-based losses operate on distributional similarity, suitable for cross-modal mapping and feature compression, but may overlook fine-grained feature fidelity.

In summary, the L1 loss demonstrates optimal performance as a supervised feature reconstruction method, owing to its robustness to outliers, its effectiveness in preserving discriminative signal details, and its stable gradient properties during optimization.

### 6.3. Enhancing ECG Signal Classification Across Different Leads

**Figure 8:**
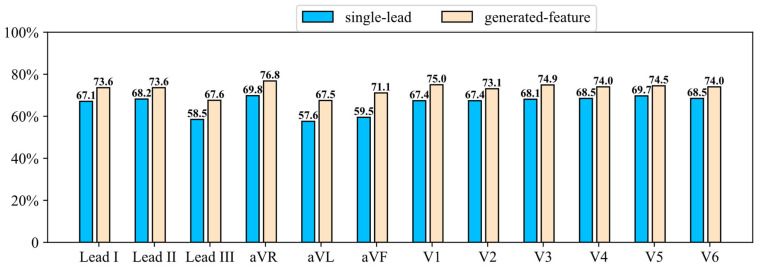
Comparison of classification performance (F1 score) across different lead inputs.

In a standard 12-lead ECG system, each lead records the electrical activity of the heart from a different spatial orientation, reflecting distinct anatomical and electrophysiological regions. Limb leads (I, II, III, aVR, aVL, aVF) mainly capture the electrical activity in the frontal plane, while precordial (chest) leads (V1–V6) provide information from the horizontal plane. Since different leads carry different information, we conduct experiments on each of the 12 leads to comprehensively evaluate the SLFR-Net, with the corresponding results shown in Figure 8.

Compared with the single-lead baseline, the SLFR-Net achieved consistent performance improvements of 0.05–0.10 across all leads. More importantly, the performance differences among leads were relatively small, indicating that the proposed framework successfully learns lead-independent latent feature representations, enabling it to adapt to inputs from different ECG leads.

Among all 12 leads, aVR performed best in both models, likely due to its unique right-superior view and low information redundancy, resulting in a richer concentration of discriminative information. Among the precordial leads, V3 and V5 achieved near-optimal performance in our model, consistent with their established clinical value in detecting left ventricular, septal, and lateral wall abnormalities. In contrast, leads III, aVL, and aVF showed the lowest performance in single-lead mode, though the feature reconstruction model partially compensated for their limitations. Overall, though the feature reconstruction strategy consistently improves single-lead-based classification performance regardless of which individual lead is used as input, the diagnostic information content and the corresponding reconstruction difficulty vary across leads due to their differing electrophysiological perspectives.

We also evaluated the performance enhancement achieved by the proposed method using 3-lead inputs (leads III, aVR, and V2 [21]) in comparison to a 3-lead model. Experimental results are shown in Table 12. The performance of the 3-lead model still showed a noticeable gap compared to the 12-lead model, whereas the 3-lead-based feature reconstruction model achieved results closely approaching those of the 12-lead model. Compared with the 3-lead model, our model achieved superior performance, with improvements exceeding 3% across nearly all evaluation metrics. Notably, the recall of our model exceeded that of the 12-lead model, reaching 0.786.

The results indicate that as the input information increases, our model achieves further performance gains, approaching or even surpassing the 12-lead model in multiple diagnostic categories, verifying the reliability of our method. This may be attributed to the fact that, compared to the complete 12-lead system, the reconstructed model benefits from reduced information redundancy, which contributes to its superior performance on specific evaluation metrics.

## 7. Conclusions

To bridge the gap in spatial information between single-lead ECGs and standard 12-lead ECGs, we propose SLFR-Net, a novel CNN–Transformer-based framework capable of reconstructing diagnostic features of 12-lead ECGs from single-lead inputs, thereby enhancing the discriminative capability of single-lead models. SLFR-Net uses a transformer-based reconstruction module to infer the missing information by aligning the single-lead representation with the feature space learned from full 12-lead ECGs. A feature-fusion mechanism is further introduced to integrate reconstructed features with original features, enabling the classifier to leverage both fine-grained morphology and broader diagnostic patterns. Experiments on the CPSC2018 and CODE-15% datasets demonstrate that the proposed method substantially outperforms the single-lead baseline, achieving notable gains in accuracy, recall, and F1-score.

Despite the promising results achieved in this study, several limitations remain and deserve further investigation. First, although the proposed method consistently improves the classification performance of single-lead ECG, a noticeable gap compared with the 12-lead model still remains. We believe that this limitation mainly arises from the inherent difference in information completeness between single-lead and 12-lead ECG. Since single-lead ECG only reflects cardiac activity from a limited perspective, it inherently contains less discriminative spatial information for classification than 12-lead ECG. As a result, single-lead methods generally fall short of the performance of 12-lead models. Although the proposed feature reconstruction and fusion strategy can compensate for the missing information to some extent and help reduce this gap, the current experimental results still indicate that the performance difference remains evident. To address this limitation, future work will focus on several directions. First, more advanced classification architectures will be explored to further improve single-lead ECG classification performance. Second, larger-scale datasets will be considered, since more data may help the model learn richer patterns and knowledge, thereby improving both representation ability and generalization performance. Third, instead of fixing the input to a specific lead, we will explore a training strategy in which different leads are randomly selected as input, which may help the model learn more general knowledge beyond a single fixed perspective.

Second, although the proposed method achieves promising results under controlled experimental conditions, direct deployment in real-world wearable-device scenarios remains challenging. To facilitate the practical application [47,48] of the proposed method in wearable devices, further optimizations in computational efficiency, model complexity, and robustness under realistic signal acquisition conditions are required. Two practical implementation routes can be considered: a local-acquisition and cloud-analysis framework, in which wearable devices collect single-lead ECG signals for cloud-based processing, or an on-device deployment strategy that reduces model complexity through pruning, quantization, and lightweight architectural design.

Third, although the proposed method achieves promising results under controlled experimental conditions, its reliability in real-world applications still needs further improvement. In practical wearable ECG scenarios, model performance may be affected by noise, motion artifacts, and other acquisition disturbances. Moreover, the model’s generalization ability across different datasets and clinical environments still requires further validation, as differences in acquisition devices, patient populations, signal quality, and labeling criteria may substantially affect performance. In addition, the current model still lacks sufficient interpretability analysis, which may limit its clinical trust and practical applicability. Therefore, future work will focus on improving robustness under realistic signal acquisition conditions, strengthening cross-database generalization, and further exploring interpretability methods such as SHAP, Grad-CAM, and attention visualization to provide more intuitive evidence for model predictions.

## Figures and Tables

**Table 1:** Tensor size changes in the main stages of the model.

Operator	Input Size	Output Size
OS-CNN	(N, 1)	(N/2, 192)
CNN Blocks	(N/2, 192)	(N/64, 1536)
Transformer	(N/64, 1536)	(N/64, 1536)
Reconstruction module	(N/64, 1536)	(N/64, 1536)
Feature fusion module	(N/64, 1536)	(N/64, 1536)
Classifier	(N/64, 1536)	(1, K)

Note: In the tensor size notation (N, C), N denotes the signal length and C denotes the number of channels. The classifier output is denoted as (1, K), where K is the number of classes.

**Table 2:** Distribution of the CPSC2018 dataset.

Type	Normal	AF	I-AVB	LBBB	RBBB	PAC	PVC	STD	STE
Recordings	896	978	669	188	1611	544	585	771	183

**Table 3:** Distribution of the CODE-15% dataset.

Type	Normal	1dAVb	RBBB	LBBB	SB	ST	AF
Recordings	30,000	4196	7685	4748	4890	6976	5609

**Table 4:** Results of feature reconstruction experiment on the CPSC2018 dataset.

Model	Acc	Pre	Rec	F1	AUC
12-lead model	0.813	0.793	0.780	0.781	0.948
Single-lead model	0.713	0.687	0.674	0.671	0.929
SLFR-Net	0.776	0.734	0.743	0.736	0.943

**Table 5:** Results of feature reconstruction experiment on the CODE-15% dataset.

Model	Acc	Pre	Rec	F1	AUC
12-lead model	0.950	0.930	0.939	0.935	0.946
Single-lead model	0.932	0.910	0.911	0.909	0.948
SLFR-Net	0.938	0.916	0.922	0.919	0.951

**Table 6:** Comparison between the SLFR-Net and classification methods based on reconstructed signals and knowledge distillation on the CPSC2018 dataset.

Methods	Acc	Pre	Rec	F1	AUC
SLFR-Net	**0.776**	**0.734**	**0.743**	**0.736**	0.943
EKGAN [37]	0.722	0.708	0.680	0.680	0.919
MCMA [44]	0.732	0.699	0.683	0.688	0.924
GAN [38]	0.737	0.732	0.691	0.700	-
KD	0.751	0.722	0.704	0.707	0.928
KD_KL	0.759	0.717	0.730	0.721	**0.944**
KD_L1	0.743	0.705	0.698	0.698	0.937

Bold values indicate the best results in each column.

**Table 7:** Comparison between the SLFR-Net and classification methods based on reconstructed signals and knowledge distillation on the CODE-15% dataset.

Methods	Acc	Pre	Rec	F1	AUC
SLFR-Net	**0.938**	**0.916**	0.922	**0.919**	**0.951**
EKGAN [37]	0.932	0.909	0.910	0.909	0.947
MCMA [44]	0.928	0.902	0.909	0.905	0.947
KD	**0.938**	**0.916**	**0.923**	**0.919**	0.950
KD_KL	**0.938**	0.915	0.922	**0.919**	0.950
KD_L1	0.937	0.915	0.921	0.918	0.950

Bold values indicate the best results in each column.

**Table 8:** Ablation experiments on each module.

Pre-Model	Feature Reconstruction	Feature Fusion	Acc	Pre	Rec	F1	AUC
			0.713	0.687	0.674	0.671	0.929
**✓**			0.742	0.701	0.701	0.699	0.935
**✓**	**✓**		0.771	0.726	0.733	0.728	0.936
**✓**	**✓**	**✓**	**0.776**	**0.734**	**0.743**	**0.736**	**0.943**

**✓** indicates that the corresponding module is included in the proposed method. Bold values indicate the best results in each column.

**Table 9:** Layers of reconstruction module.

Layers	Acc	Pre	Rec	F1	AUC
1	0.763	0.711	0.727	0.717	0.936
2	**0.776**	**0.734**	**0.743**	**0.736**	**0.943**
3	0.765	0.718	0.737	0.725	0.940
4	0.766	0.718	0.731	0.723	0.935

Bold values indicate the best results in each column.

**Table 10:** Classification performance using different reconstruction frameworks.

Reconstruction Framework	Acc	Pre	Rec	F1	AUC
CNN	**0.779**	**0.736**	0.736	0.733	0.924
Transformer	0.776	0.734	**0.743**	**0.736**	**0.943**

Bold values indicate the best results in each column.

**Table 11:** Classification performance using different loss functions.

Loss Functions	Acc	Pre	Rec	F1	AUC
COS	0.764	0.709	0.724	0.715	0.936
L1	**0.776**	**0.734**	**0.743**	**0.736**	**0.943**
L2	0.751	0.7	0.717	0.706	0.941
KL	0.760	0.717	0.728	0.721	0.940
JS	0.768	**0.734**	0.735	0.733	**0.943**

Bold values indicate the best results in each column.

**Table 12:** Results of 3-lead reconstruction experiment.

Model	Acc	Pre	Rec	F1	AUC
12-lead model	0.813	0.793	0.780	0.781	0.948
3-lead model	0.771	0.733	0.751	0.739	0.952
Our model (3-lead inputs)	0.806	0.763	0.786	0.773	0.950

## Data Availability

The data used in the article was from the public database. The CPSC2018 dataset and the CODE-15% dataset used in this work are publicly available from their corresponding official sources.
